# Human Personality Is Associated with Geographical Environment in Mainland China

**DOI:** 10.3390/ijerph191710819

**Published:** 2022-08-30

**Authors:** Liang Xu, Yanyang Luo, Xin Wen, Zaoyi Sun, Chiju Chao, Tianshu Xia, Liuchang Xu

**Affiliations:** 1 Department of Psychology, College of Education, Zhejiang University of Technology, Hangzhou 310014, China; 2 Department of Psychology and Behavioral Sciences, Zhejiang University, Hangzhou 310058, China; 3 Department of Information Art and Design, Tsinghua University, Beijing 100084, China; 4 Financial Big Data Research Institute, Sunyard Technology Co., Ltd., Hangzhou 310053, China; 5 College of Mathematics and Computer Science, Zhejiang A&F University, Hangzhou 311300, China; 6 College of Computer Science and Technology, Zhejiang University, Hangzhou 310063, China

**Keywords:** Big Five personality, geographical environment, mountainousness, cultural differences, multilevel modelling, machine learning

## Abstract

Recent psychological research shown that the places where we live are linked to our personality traits. Geographical aggregation of personalities has been observed in many individualistic nations; notably, the mountainousness is an essential component in understanding regional variances in personality. Could mountainousness therefore also explain the clustering of personality-types in collectivist countries like China? Using a nationwide survey (29,838 participants) in Mainland China, we investigated the relationship between the Big Five personality traits and mountainousness indicators at the provincial level. Multilevel modelling showed significant negative associations between the elevation coefficient of variation (*Elevation CV*) and the Big Five personality traits, whereas mean elevation (*Elevation Mean*) and the standard deviation in elevation (*Elevation STD*) were positively associated with human personalities. Subsequent machine learning analyses showed that, for example, *Elevation Mean* outperformed other mountainousness indicators regarding correlations with neuroticism, while *Elevation CV* performed best relative to openness models. Our results mirror some previous findings, such as the positive association between openness and *Elevation STD*, while also revealing cultural differences, such as the social desirability of people living in China’s mountainous areas.

## 1. Introduction

There are numerous accounts of the personality traits of individuals who live in different parts of China, with some inhabitants being described as pleasant and naïve and others as rude and pushy [1]. Geographical variation of personality traits has been observed in many nations including the United States [2,3,4], Switzerland [5], the Russian Federation [6], and Great Britain [7,8]. To interpret the geographical differences in human personality, researchers have looked into various possible mechanisms, such as climate [9], selective migration [10], sociocultural legacies [11], and physical topography [2]. As a core feature of physical topography, mountainousness has shown association with a variety of personality characteristics in the United States [2], which drew our attention.

Before studying geographic differences in personality, we first need to determine the personality model to be used. Since personality traits are typically described using words [12], various personality models, such as the HEXACO model [13], the sixteen primary factors [14], and the Big Five taxonomy [15], have been proposed by extracting the common factors of a huge number of personality descriptors. Among them, the Big Five model (also known as the five-factor model) is the most well studied and cross-culturally applicable model [16]; it has been widely used in research on geographical psychology [2,7,8,9]. Following the work of Goldberg [15], five factors have been extensively tested: (1) agreeableness (tendency to be likeable and pleasant to satisfy others) [17], (2) extraversion (tendency to experience, exhibit, and enjoy positive affect, social attention, assertive behavior, potential rewards, and so on) [18,19,20], (3) conscientiousness (tendency to obey socially mandated norms, to be goal-oriented, to plan, and to defer gratification) [21], (4) neuroticism (tendency to experience negative emotions) [22], and (5) openness to experience (tendency to be inquisitive, inventive, and unconventional) [23]. Hence, the Big Five personality taxonomy was used in the present study.

Since mountainous locations are often inhospitable and environmentally severe, the inhabitants who live in such settings may leave an indelible mark on their characteristics [11,24]. So why is it possible that mountainousness influences the geographic distribution of personality traits? Götz et al. identified two possible reasons [2]. One is historical, i.e., mountainous environments have traditionally drawn a unique set of individuals who valued the freedom that the nature offers and were ready to be apart from people and things from the past [11,25]. The other is that individuals became cautious and not pro-social because of the harsh environment, which forces them to do risky things to ensure their survival [26]. In fact, more clues appear when we look at the Big Five personality traits independently. For example, Oishi and colleagues found that introverts favor secluded and hilly areas whereas extraverts prefer flat and open regions [27]. According to work by Plaut et al., residents of mountain regions are more open-minded and curious [28], associating with openness to experience. Regarding neuroticism, residents of mountainous areas are less worried and anxious [28], and individuals who are self-reliant and emotionally stable are more likely to thrive in mountainous regions [29]. Following upon these mixed findings, Götz et al. conducted a data-driven study in USA and found that individuals in mountainous regions scored lower on agreeableness, extraversion, neuroticism, and conscientiousness but higher on openness to experience [2].

The majority of such research, however, has been done in individualist countries. As such, we sought to determine whether it was possible to reproduce the above findings in a collectivist nation. In individualist societies, people are autonomous and self-contained from their in-group [30]. They prioritize personal aims above in-group goals and act largely on the basis of their attitudes rather than in-group norms [30]. Previous works have shown that individualism is linked to personality traits, revealing, for example, a negative association with agreeableness [31,32] and conscientiousness [33]. Disparities in personality may be caused by cultural differences between collectivism and individualism [34], which may also alter the underlying factors that influence personality [35]. Thus, we would like to investigate the relationships between mountainousness and personality in China, a representative collectivist society [36]. China is geographically large enough to conduct our investigation. Furthermore, a previous study explored the relationship between climate and personality in the nation [9], providing an important reference for this work. In addition, China’s terrain, unlike that of many other countries, is high in the west and low in the east, with a ladder-like distribution [37]. The stereotype is that Chinese mountain residents are straightforward, honest, and trustworthy [38]. We believe that China’s special terrain and culture can lead to interesting and different discoveries.

A previous work defined mountainousness based on two components—elevation and hilliness [39]. Elevation refers to altitude, and hilliness describes the slope and shape of a region. Considering this distinction, Götz et al., used the mean elevation (*Elevation Mean*) as an indicator of overall altitude and the standard deviation of elevation (*Elevation STD*) and the mean squared successive difference of elevation (*Elevation MSSD*) as indicators of hilliness [2]. As such, *Elevation Mean* and *Elevation STD* were used in this study. However, *Elevation MSSD* was excluded, because our work was conducted at a province level. Instead, we added a new indicator—the elevation coefficient of variation (*Elevation CV*)—which has been widely used to describe terrain relief [40,41].

In summary, the present study used a data-driven approach to re-examine the relationship between mountainousness and personality traits in a collectivist nation—China. Our goal was to determine whether China’s unique geographical environment affects the regional clustering of personality traits. Using a sample of 29,838 participants, we investigated associations among the Big Five personality traits and mountainousness indicators across twenty-two provinces, five autonomous regions, and four municipalities in Mainland China. From a cross-cultural perspective, this research complements previous findings and may serve as a useful reference for future geographic psychology research. From a practical perspective, regional aggregated data describing personality traits may be a valuable resource for the government in terms of developing regionalization strategies and assisting various regions in the areas of economics, culture, health, and so forth.

## 2. Methods

In this section, three aspects are introduced. First, Section 2.1 describes how the individual factors, including the Big Five personality traits and demographic information (e.g., sex, age, and education), were collected using a nationwide survey. Second, we present our objective measurements of mountainousness in Section 2.2. Finally, two data analysis methods, i.e., multi-level modelling and random forests, are introduced in Section 2.3.

### 2.1. Individual Factors

We used data from the China Family Panel Studies (CFPS), a public database collected by the Institute of Social Science Survey at Beijing University [40], to achieve our goals. CFPS is a nationwide, biennial survey of Chinese families, which contains information about demographics, geographic location, subjective attitudes, personality, assets, incomes, and so forth [42]. We applied the CFPS 2018 database (Accessible at https://www.isss.pku.edu.cn/cfps, accessed on 30 July 2022), including 37,354 individuals across twenty-two provinces, five autonomous regions, and four municipalities in Mainland China. Since autonomous regions and municipalities are considered to be at the same level as provinces in China, we describe them here as “provinces” for the sake of convenience.

Demographic information from the CFPS 2018 database, including sex, age (converted from birth year), and education level (from 1—nursery to 8—doctorate degree), were directly used in this study. In terms of geographic location, county and city information was encrypted, so only the province information for each individual was used. For each province, the mean values of latitude and longitude were calculated for subsequent data analyses, and the geographic location of each individual was matched with mountainous indicators. For personality, the CFPS 2018 used a brief 15-item version of the Big Five personality scale [42] which has been widely used in previous studies [43,44,45]. After removing samples with missing data, the individual information of 29,838 participants (48.50 ± 16.83 years old, 50.19% females) was used in subsequent modeling and analyses.

### 2.2. Mountainousness Indicators

**Figure 1:**
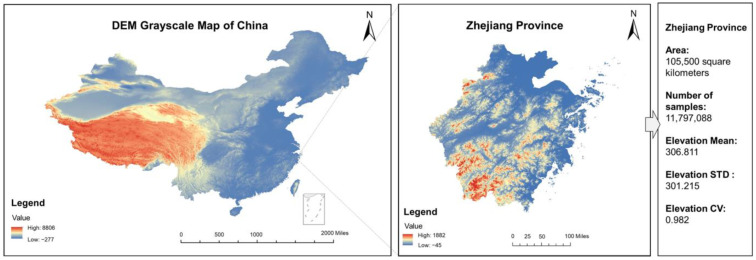
Calculation of provincial mountainousness indicators.

To determine the degree of mountainousness of various locations, we considered three indicators: the mean elevation (*Elevation Mean*), the standard deviation in elevation (*Elevation STD*), and the elevation coefficient of variation (*Elevation CV*). The original geographical data were downloaded from ASTER Global Digital Elevation Map (Accessible at https://asterweb.jpl.nasa.gov/gdem.asp, accessed on 30 July 2022) and processed using the ArcGIS program [46,47]. As shown in Figure 1, the Digital Elevation Model (DEM) is a quantitative representation of the earth’s surface, providing basic information about terrain relief [48]. To achieve our goals, we first sampled the elevation values for each 30 × 30 square meter parcel in each province using a DEM grayscale map. We then calculated the mountainousness indicators as follows: *Elevation Mean* is the average of all the sampled values; *Elevation STD* is the standard deviation of the sampled values; and *Elevation CV* is the ratio of *Elevation STD* to *Elevation Mean*. For instance, as shown in Figure 1, we collected 11,797,088 elevation values for Zhejiang Province’s 105,500 square kilometer area. Through a series of calculations, we came to the conclusion that the *Elevation Mean* is 306.811, *Elevation STD* is 301.215, and *Elevation CV* is 0.982 in this province. Finally, the calculated provincial mountainous indicators were matched to individual locations.

### 2.3. Data Analysis

To meet our research goals, we used a two-pronged analysis strategy, similar to that used by Götz and colleagues [2]. To begin, we used multi-level modeling to investigate the impacts of mountainousness on personality traits (see Section 2.3.1). Complementing the multilevel modelling, we then used supervised machine learning to assess the explanatory power of three mountainousness indicators (see Section 2.3.2).

#### 2.3.1. Multilevel Modeling

Multilevel modelling was first conducted with the 29,838 samples. Based upon the methods applied in works with similar data structures [2,9,49], we constructed random-intercept-fixed-slope multilevel models. For each combination of Big Five personality traits and mountainousness indicators (5 × 3), we built four models as follows: (1) using only the individual-level variables (age, sex, and education); (2) using the individual-level variables and two macro-environmental variables (latitude and longitude); (3) using the individual-level variables and the target indicator of mountainousness; and (4) using all the variables to predict personality traits (for examples, see Supplementary Materials Tables S1–S5). The individual-level and macro-environmental variables were treated as Level 1 and Level 2 control variables, respectively. We present standardized betas of fixed coefficients with 95% confidence intervals for ease of interpretation [50]. The information criteria indices include Akaike information criterion and Bayesian information criterion [51], which are also reported.

#### 2.3.2. Random Forests Analyses

The second step of our dualist approach was to use data-driven machine learning analyses to determine the feature importance of our predictors. This study relied on random forests (RF), a traditional machine learning technique which has been widely used in previous works for similar purposes [52,53]. The RF technique combines predictions from a variety of decision trees which are built by repeatedly pulling bootstrap samples from the original data [2,52]. The feature importance, as determined by the decision trees, can reveal nonlinear relationships among the model inputs (e.g., age, sex, and education) and the ground truth (personality traits).

In this study, we used all the predictor variables (i.e., individual factors, environmental variables, and mountainousness indicators) as model inputs, the Big Five personality traits as ground truth data, and the RF algorithm to construct regressions for each personality trait. A grid parameter search was applied to find the best parameters for our models (the results are presented in Supplementary Materials Table S6). The explanatory power of the inputs for each regression was then calculated based on the Gini importance [52]. To prevent overfitting, we used the tenfold cross-validation technique, which uses 90% of the data to train the models and the remaining instances as testing data [53]. Hence, the feature importance was also calculated ten times for each personality trait. Notably, the absolute score of feature importance has no meaning on its own [2], and the primary goal of the outcome in this step is to obtain a relative ranking and comparison of the predictor variables.

## 3. Results

### 3.1. Distribution of Personality

**Figure 2:**
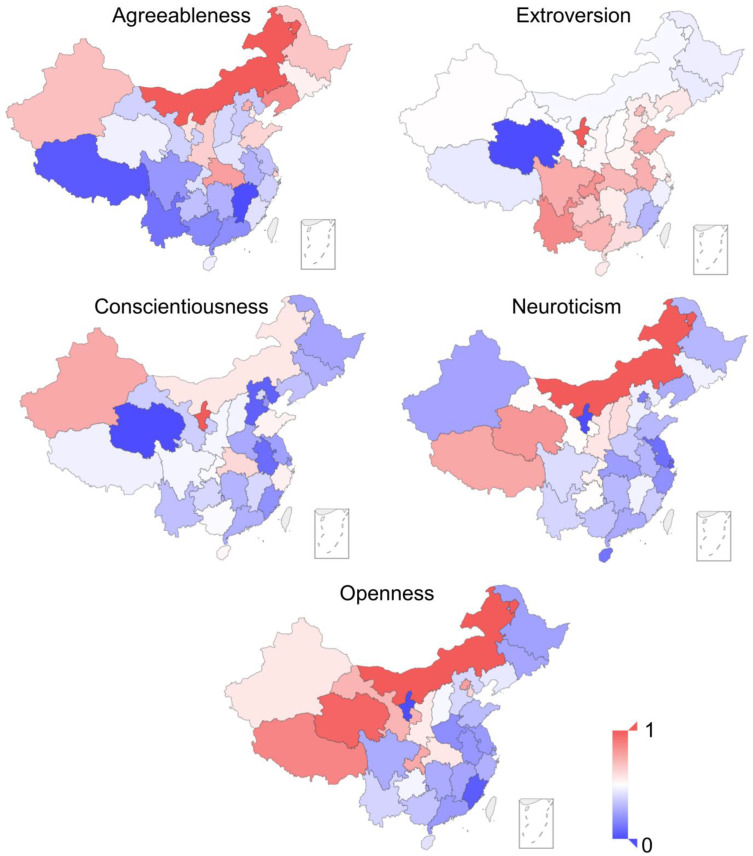
Distribution of Big Five personality traits in Mainland China (max–min scaling).

As a first step, we investigated whether there were differences in the Big Five personality scores among provinces. The Kruskal Wallis tests showed that all the five personality traits were significantly different in different provinces: agreeableness (χ^2^(30) = 189.361, *p* < 0.001), extraversion (χ^2^(30) = 275.607, *p* < 0.001), conscientiousness (χ^2^(30) = 380.331, *p* < 0.001), neuroticism (χ^2^(30) = 451.036, *p* < 0.001), and openness (χ^2^(30) = 382.103, *p* < 0.001). The distribution of personality scores is presented in Figure 2; the personality values were scaled to a range of 0 to 1 via min–max scaling [54] to better visualize distinctions among provinces. We observed that, for example, the openness scores for the northwestern Chinese provinces were higher than those of other provinces, and that agreeableness scores were higher in northern provinces than in southern provinces. The mechanism underlying such geographical personality variations was investigated via subsequent data analyses.

### 3.2. Results from Multilevel Modelling

In general, the Big Five personality traits were found to be significantly linked with indicators of mountainousness. As shown in Table 1, multi-level modelling showed that the elevation coefficient of variation (*Elevation CV*) had negative associations with agreeableness (*β*[95% confidence interval (CI)] = −0.3862[−0.4414, −0.3309], *p* < 0.001), extraversion (*β*[95% CI] = −0.2662[−0.3313, −0.2012], *p* < 0.001), conscientiousness (*β*[95% CI] = −0.4951[−0.5552, −0.4349], *p* < 0.001), neuroticism (*β*[95% CI] = −0.6180[−0.6841, −0.5519], *p* < 0.001), and openness (*β*[95% CI] = −0.6854[−0.7619, −0.6089], *p* < 0.001). The mean elevation (*Elevation Mean*) was positively associated with agreeableness (*β*[95% CI] = 0.0005[0.0005, 0.0006], *p* < 0.001), extraversion (*β*[95% CI] = 0.0006[0.0005, 0.0006], *p* < 0.001), conscientiousness (*β*[95% CI] = 0.0006[0.0006, 0.0007], *p* < 0.001), neuroticism (*β*[95% CI] = 0.0007[0.0006, 0.0007], *p* < 0.001), and openness (*β*[95% CI] = 0.0007[0.0007, 0.0008], *p* < 0.001) (see Supplementary Materials Table S7). The standard deviation in elevation (*Elevation STD*) had positive associations with agreeableness (*β*[95% CI] = 0.0011[0.0010, 0.0011], *p* < 0.001), extraversion (*β*[95% CI] = 0.0012[0.0011, 0.0013], *p* < 0.001), conscientiousness (*β*[95% CI] = 0.0013[0.0012, 0.0013], *p* < 0.001), neuroticism (*β*[95% CI] = 0.0013[0.0012, 0.0014], *p* < 0.001), and openness (*β*[95% CI] = 0.0014[0.0013, 0.0015], *p* < 0.001) (see Supplementary Materials Table S8), which contradicts previous research [5]. To our surprise, *Elevation Mean* had positive associations with all five personality traits, in contrast to previous findings in individualistic nations (excluding openness) [2]. The new indicator, *Elevation CV*, showed good prediction effects for all the five personality traits and deserved further study. Hence, we next used nonlinear analysis to re-examine the above findings.

### 3.3. Results from Random Forests Analyses

**Figure 3:**
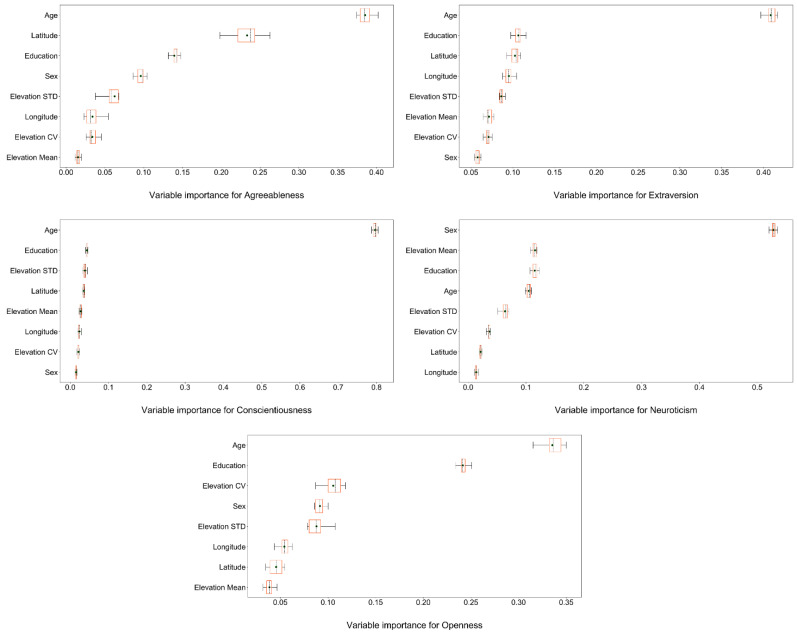
Degrees of importance of variables for the Big Five personality traits. The black dots indicate mean values, the black dotted lines indicate median values, and the boxplots are sorted by the mean values.

**Figure 4:**
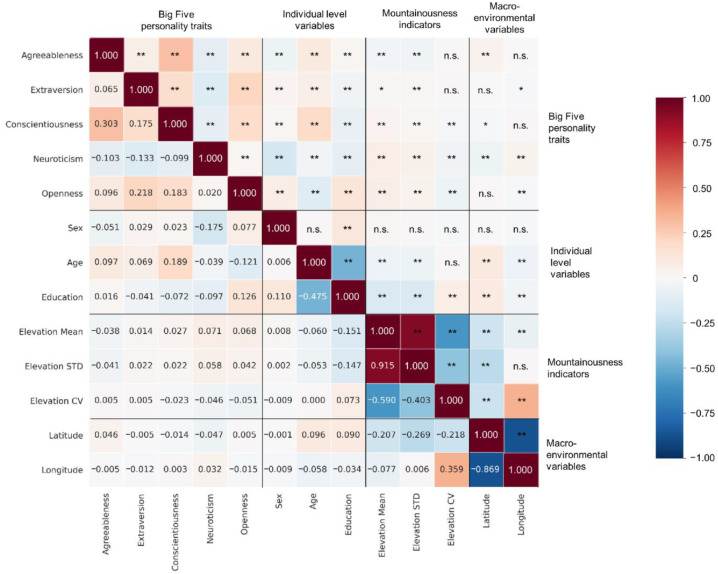
Zero-order correlations among variables. The following statistical significance levels of the given correlations are indicated in the upper triangle: * *p* < 0.05, ** *p* < 0.01; n.s. = not significant.

The multilevel modeling results were then complemented by RF analysis, which corroborated the findings presented in Section 3.2. As shown in Figure 3, RF analyses showed that mountainousness indicators are significant predictors of personality. For agreeableness, *Elevation STD* (accounting for 6.26% of the models) was the most important mountainousness indicator, whereas *Elevation Mean and Elevation CV* showed low predictive effects, accounting for 1.54% and 3.39% of the models, respectively. In terms of extraversion, *Elevation CV* was the most important mountainousness indicator (8.53%), followed by *Elevation STD* (6.87%) and *Elevation Mean* (6.47%). For conscientiousness, age accounted for 79.61% of the models, and all mountainousness indicators showed low predictive effects (*Elevation STD*: 3.84%; *Elevation CV*: 2.10%; and *Elevation Mean*: 2.71%). Regarding neuroticism, unlike other personality traits, *Elevation Mean* was the most significant mountainousness indicator (11.56%), followed by *Elevation STD* (6.36%) and *Elevation CV* (3.72%). Finally, the result of the openness model was similar to that of extroversion; that is, *Elevation CV* was the most important mountainousness indicator (10.54%), followed by *Elevation STD* (8.79%) and *Elevation Mean* (3.85%). In sum, *Elevation STD* was particularly associated with agreeableness, *Elevation CV* was strongly associated with extraversion and openness, and *Elevation Mean* was strongly associated with neuroticism. The above findings were corroborated by the results from zero-order correlation analyses (see Figure 4). For examples, Steiger’s Z tests [55] showed that the zero-order correlation of neuroticism with *Elevation Mean* was stronger than that with other mountainousness indicators (*Elevation STD*: Z = 5.458, *p* < 0.001; *Elevation CV*: Z = 11.347, *p* < 0.001), while the zero-order correlation of agreeableness with *Elevation STD* was stronger than that with *Elevation CV* (Z = 4.727, *p* < 0.001).

## 4. Discussion

The present work employed advanced analysis approaches to re-examine whether personality traits are linked to degree of mountainousness in Mainland China. A two-pronged analyses showed significant associations between the Big Five personality traits and mountainousness indicators across multilevel modelling and RF techniques. In general, the elevation coefficient of variation (*Elevation CV*), which describes terrain relief [40], showed negative associations with all Big Five personality traits, whereas, the mean elevation (*Elevation Mean*) and the standard deviation in elevation (*Elevation STD*) were positively associated with personality traits.

Our findings mirror some of the insights from previous research [2,5,56], but differences also exist. In terms of similarities, we all observed positive associations of openness with *Elevation Mean* and *Elevation STD.* Individuals who move from the luxuries of civilization to harsh terrains may have to face unforeseen obstacles and experiences [24]. Openness to experience might be necessary for mastering the difficult ecological circumstances of life in a mountainous region [26]. Hence, cross-cultural consistency regarding openness was to be expected. Although openness is often treated as a characteristic of individualism [31], it is also associated with the motivation to pursue goals that are impossible to achieve in certain environments [57]. The mindsponge theory [58] can also help explain the cross-cultural consistency. In an increasingly interconnected society, the mindsponge assists people in determining if it is worthwhile to enable cross-cultural ideals to enter their “comfort zone”, i.e., the “nucleus” of their psyche. The consistent outcomes of openness might be considered an acculturation phenomenon, reflecting the affiliation of some Chinese people with certain Western values.

In terms of the differences, two major aspects need to be discussed. First, previous research found that *Elevation STD* was the most effective mountainousness indicator, outperforming others (i.e., mean squared successive difference in elevation and mean elevation) for all personality traits [2]. In the present study, this phenomenon was not so clear. For example, *Elevation Mean* outperformed *Elevation STD* for predicting neuroticism, but the opposite was true in the agreeableness and extroversion models (see Figure 3). The essence of the distinction is that the altitude has a significant impact upon certain personality traits in Mainland China. Unlike mountainous regions in the United States, which straddle the North American continent, China is located in the east of the Asian continent, resulting in an association between coastline distance and altitude. Hence, one possible explanation is that coastline distance in Mainland China may influence personality. Previous research found that mountain lovers were more introverted than ocean lovers, and when it came to socializing, people were more likely to choose the ocean over the mountains [27]. People who live near the sea and those who live inland may have different personalities, which may contribute to the statistical association between altitude and personality.

Another major aspect is the diametrically opposed relationship between *Elevation STD* and personalities in different studies. For example, previous studies have found negative associations between agreeableness and *Elevation STD* in many nations, such as the United States [2] and Switzerland [5], while agreeableness was positively associated with *Elevation STD* in China, revealing cultural difference. In fact, traditionally, a great deal of Chinese art (poetry, literature, songs) extols the virtues of people living in mountainous areas [59,60]. Social desirability and the tendency for people to present themselves in a generally favorable fashion [61] may have made the populations in mountainous areas more agreeable, enthusiastic, and kind-hearted. As a result, the mountainous areas in Mainland China showed higher agreeableness, extraversion, and conscientiousness values.

Our work is also of practical value. Previous works have shown that regional personality characteristics are linked to a variety of economic, political, health, and social factors [4,62,63]. Hence, regional personality variations can assist us in developing specific strategies to aid regional development. For examples, since regional levels of openness and conscientiousness are positively associated with economic prosperity and resilience [63], liberal policies and innovation may be more appropriate in areas with higher proportions of creative and industrious individuals. Additionally, high levels of neuroticism are linked to heart disease, mental health problems, and cancer [4], so we should provide more psychological support in regions with higher levels of this trait. Governments and researchers could customize strategies for different areas in China based on our findings.

This study has several notable limitations. First, it investigated the association between personality traits and mountainousness indicators at the province level. Although other studies have been conducted at the province (or state) level [64], more granular regions, such as cities [9] or Zip codes [2,7], may yield more reliable results. To protect the privacy of participants, the CFPS encrypted the geographic information about districts, counties, and cities [42]. Future, more in depth research on geographical environments, with participants’ permission, is planned. Second, the CFPS used a brief, 15-item version of the Big Five personality test [42] to measure personality, likely making it less reliable than other Big Five personality inventories with more items, such as the 44-item [65] and 240-item [66] tests. Although administering a long-form test to a big sample is difficult, we believe that future research will be able to employ more effective scales. Third, our methodology design was based on previous works, so its theoretical contribution is limited. Fourth, this study did not consider the impact of co-existing variables on personality. Cultural additivity [67] and transmission through ancient works [68] may be essential study avenues in the future. Finally, using a data-driven approach, we interpreted geographical differences in human personality according to mountainousness; more mechanisms, including selective migration [10], sociocultural legacies [11], and so forth, need to be investigated and re-examined in future research.

## 5. Conclusions

In conclusion, this study explored regional variances in the personalities of residents of Mainland China by examining the associations between Big Five personality traits and mountainousness indicators. Using a two-pronged strategy to analyze data from 29,838 individuals, we found negative associations between *Elevation CV* and Big Five personality traits, and positive associations among *Elevation Mean*, *Elevation STD*, and personalities. A RF analyses showed that *Elevation STD* was particularly associated with agreeableness, *Elevation CV* was strongly associated with extraversion and openness, and *Elevation Mean* was strongly associated with neuroticism. These findings mirrored some previous discoveries and revealed cultural differences between China and individualistic nations. Our study complements previous findings from a cross-cultural perspective and provides an important reference for future geographic psychology research.

## Figures and Tables

**Table 1:** Results from multilevel modelling for the elevation coefficient of variation.

Predictor	Agreeableness	Extraversion	Conscientiousness	Neuroticism	Openness
	*β* (*p*) [95% CI]	*β* (*p*) [95% CI]	*β* (*p*) [95% CI]	*β* (*p*) [95% CI]	*β* (*p*) [95% CI]
Sex	−0.1215 (<0.001) [−0.1652, −0.0777]	0.2333 (<0.001) [0.1818, 0.2848]	0.1805 (<0.001) [0.1329, 0.2281]	−0.6071 (<0.001) [−0.6594, −0.5548]	0.4529 (<0.001) [0.3923, 0.5134]
Age	0.0232 (<0.001) [0.0217, 0.0247]	0.0193 (<0.001) [0.0176, 0.0211]	0.0358 (<0.001) [0.0342, 0.0374]	−0.0013 (=0.136) [−0.0031, 0.0004]	−0.0021 (=0.044) [−0.0041, −0.0001]
Education	0.1797 (<0.001) [0.1619, 0.1976]	0.0794 (<0.001) [0.0584, 0.1004]	0.1442 (<0.001) [0.1248, 0.1636]	−0.0911 (<0.001) [−0.1124, −0.0698]	0.2448 (<0.001) [0.2201, 0.2695]
Latitude	0.0638 (<0.001) [0.0627, 0.0649]	0.0545 (<0.001) [0.0532, 0.0558]	0.0565 (<0.001) [0.0553, 0.0577]	0.0585 (<0.001) [0.0572, 0.0598]	0.0559 (<0.001) [0.0544, 0.0574]
Longitude	0.0717 (<0.001) [0.0709, 0.0726]	0.0634 (<0.001) [0.0624, 0.0644]	0.0695 (<0.001) [0.0685, 0.0704]	0.0720 (<0.001) [0.0710, 0.0730]	0.0654 (<0.001) [0.0643, 0.0666]
Elevation CV	−0.3862 (<0.001) [−0.4414, −0.3309]	−0.2662 (<0.001) [−0.3313, −0.2012]	−0.4951 (<0.001) [−0.5552, −0.4349]	−0.6180(<0.001) [−0.6841, −0.5519]	−0.6854 (<0.001)
					[−0.7619, −0.6089]
**Model fit statistics**					
AIC	123,465	133,181	128,506	134,112	142,825
BIC	123,531	133,248	128,573	134,179	142,892

Elevation CV: The elevation coefficient of variation; AIC: Akaike information criterion; BIC: Bayesian information criterion.

## Data Availability

The CFPS data is available at https://www.isss.pku.edu.cn/cfps (accessed on 30 July 2022), and the geographical data from ASTER Global Digital Elevation Map is available at https://asterweb.jpl.nasa.gov/gdem.asp (accessed on 30 July 2022).

## Supplementary Materials

The following are available online at https://www.mdpi.com/article/10.3390/ijerph191710819/s1, Tables S1–S5: Results from multilevel modelling for Big Five personality traits and the elevation coefficient of variation, Table S6: Results of parameter search for the prediction models, Table S7: Results from multilevel modelling for the mean elevation, Table S8: Results from multilevel modelling for the standard deviation in elevation.

## References

[1] Rentfrow P.J. (2020). Geographical Psychology. Curr. Opin. Psychol..

[2] Götz F.M., Stieger S., Gosling S.D., Potter J., Rentfrow P.J. (2020). Physical Topography Is Associated with Human Personality. Nat. Hum. Behav..

[3] Elleman L.G., Condon D.M., Russin S.E., Revelle W. (2018). The Personality of U.S. States: Stability from 1999 to 2015. J. Res. Pers..

[4] Rentfrow P.J., Gosling S.D., Jokela M., Stillwell D.J., Kosinski M., Potter J. (2013). Divided We Stand: Three Psychological Regions of the United States and Their Political, Economic, Social, and Health Correlates. J. Pers. Soc. Psychol..

[5] Götz F.M., Ebert T., Rentfrow P.J. (2018). Regional Cultures and the Psychological Geography of Switzerland: Person–Environment–Fit in Personality Predicts Subjective Wellbeing. Front. Psychol..

[6] Allik J., Realo A., Mõttus R., Pullmann H., Trifonova A., McCrae R.R., Yurina A.A., Shebanets E.Y., Fadina A.G., Tikhonova E.V. (2009). Personality Traits of Russians from the Observer’s Perspective. Eur. J. Pers..

[7] Jokela M., Bleidorn W., Lamb M.E., Gosling S.D., Rentfrow P.J. (2015). Geographically Varying Associations between Personality and Life Satisfaction in the London Metropolitan Area. Proc. Natl. Acad. Sci. USA.

[8] Rentfrow P.J., Jokela M., Lamb M.E. (2015). Regional Personality Differences in Great Britain. PLoS ONE.

[9] Wei W., Lu J.G., Galinsky A.D., Wu H., Gosling S.D., Rentfrow P.J., Yuan W., Zhang Q., Guo Y., Zhang M. (2017). Regional Ambient Temperature Is Associated with Human Personality. Nat. Hum. Behav..

[10] Jokela M. (2009). Personality Predicts Migration within and between U.S. States. J. Res. Pers..

[11] Kitayama S., Conway L.G., Pietromonaco P.R., Park H., Plaut V.C. (2010). Ethos of Independence across Regions in the United States: The Production-Adoption Model of Cultural Change. Am. Psychol..

[12] Roivainen E. (2015). Personality Adjectives in Twitter Tweets and in the Google Books Corpus. An Analysis of the Facet Structure of the Openness Factor of Personality. Curr. Psychol..

[13] Ashton M.C., Lee K. (2007). Empirical, Theoretical, and Practical Advantages of the HEXACO Model of Personality Structure. Pers. Soc. Psychol. Rev..

[14] Cattell R.B. (1966). Validation and Intensification of the Sixteen Personality Factor Questionnaire. Read. Clin. Psychol..

[15] Goldberg L.R. (1990). An Alternative “Description of Personality”: The Big-Five Factor Structure. J. Pers. Soc. Psychol..

[16] De Raad B., Mlačić B., Widiger T.A. (2017). The lexical foundation of the Big-Five factor model. The Oxford Handbook of the Five Factor Model.

[17] Caspi A., Roberts B.W., Shiner R.L. (2005). Personality Development: Stability and Change. Annu. Rev. Psychol..

[18] Ashton M.C., Lee K., Paunonen S.V. (2002). What Is the Central Feature of Extraversion? Social Attention versus Reward Sensitivity. J. Pers. Soc. Psychol..

[19] Buss D.M. (1991). Evolutionary personality psychology. Annu. Rev. Psychol..

[20] Lucas R.E., Diener E., Grob A., Suh E.M., Shao L. (2000). Cross-Cultural Evidence for the Fundamental Features of Extraversion. J. Pers. Soc. Psychol..

[21] Roberts B.W., Jackson J.J., Fayard J.V., Edmonds G., Meints J., Leary M.R., Hoyle R.H. (2009). Conscientiousness. Handbook of Individual Differences in Social Behavior.

[22] Widiger T.A., Leary M.R., Hoyle R.H. (2009). Neuroticism. Handbook of Individual Differences in Social Behavior.

[23] McCrae R.R., John O.P. (1992). An Introduction to the Five-Factor Model and Its Applications. J. Pers..

[24] Kitayama S., Ishii K., Imada T., Takemura K., Ramaswamy J. (2006). Voluntary Settlement and the Spirit of Independence: Evidence from Japan’s “Northern Frontier”. J. Personal. Soc. Psychol..

[25] Olsson O., Paik C. (2016). Long-Run Cultural Divergence: Evidence from the Neolithic Revolution. J. Dev. Econ..

[26] Sng O., Neuberg S.L., Varnum M.E.W., Kenrick D.T. (2018). The Behavioral Ecology of Cultural Psychological Variation. Psychol. Rev..

[27] Oishi S., Talhelm T., Lee M. (2015). Personality and Geography: Introverts Prefer Mountains. J. Res. Personal..

[28] Plaut V.C., Markus H.R., Lachman M.E. (2002). Place Matters: Consensual Features and Regional Variation in American Well-Being and Self. J. Personal. Soc. Psychol..

[29] Ciani A.C., Capiluppi C. (2011). Gene Flow by Selective Emigration as a Possible Cause for Personality Differences between Small Islands and Mainland Populations. Eur. J. Pers..

[30] Triandis H.C. (2001). Individualism-Collectivism and Personality. J. Personal..

[31] Dollinger S.J., Preston L.A., O’Brien S.P., DiLalla D.L. (1996). Individuality and Relatedness of the Self: An Autophotographic Study. J. Personal. Soc. Psychol..

[32] Grimm S.D., Church A.T., Katigbak M.S., Reyes J.A.S. (1999). Self-Described Traits, Values, and Moods Associated with Individualism and Collectivism: Testing I-C Theory in an Individualistic (U.S.) and a Collectivistic (Philippine) Culture. J. Cross-Cult. Psychol..

[33] Moorman R.H., Blakely G.L. (1995). Individualism-Collectivism as an Individual Difference Predictor of Organizational Citizenship Behavior. J. Organiz. Behav..

[34] Kajonius P., Mac Giolla E. (2017). Personality Traits across Countries: Support for Similarities Rather than Differences. PLoS ONE.

[35] Church A.T., Lonner W.J. (1998). The Cross-Cultural Perspective in the Study of Personality: Rationale and Current Research. J. Cross-Cult. Psychol..

[36] Steele L.G., Lynch S.M. (2013). The Pursuit of Happiness in China: Individualism, Collectivism, and Subjective Well-Being During China’s Economic and Social Transformation. Soc. Indic. Res..

[37] Zhang X. Distribution of Mountains and Hills in China. https://www.osgeo.cn/post/2c1f8.

[38] Zhang H., An G., Zhao W. (2010). The Stereotyped Impression of the Characteristics of Chinese Residences at the Provincial Level from the Perspective of Regional Psychology. J. Longdong Univ..

[39] Schuler M., Stucki E., Roque O., Perlik M. Mountain Areas in Europe: Analysis of Mountain Areas in EU Member States, Acceding and Other European Countries. https://infoscience.epfl.ch/record/113427.

[40] Yang X., Wang P., Li X., Xie C., Zhou B., Huang X. (2019). Application of Topographic Slope and Elevation Variation Coefficient in Identifying the Motuo Active Fault Zone. Seismol. Egology.

[41] Zhang H., Wang X., Yu Z. (2009). Slop Surface Complexity Factor Extract and Analysis Based on ArcGIS. J. Huazhong Norm. Univ. Nat. Sci..

[42] Wu Q., Dai L., Zhen Q., Gu L., Wang Y. User Guide for China Family Panel Studies 2018. Institute of Social Science Survey, Peking University. https://www.isss.pku.edu.cn/cfps/docs/20220302153921616729.pdf.

[43] (2001). The German Socio-Economic Panel (GSOEP) after More than 15 Years—Overview. Vierteljahrsh. Zur Wirtsch..

[44] Hahn E., Gottschling J., Spinath F.M. (2012). Short Measurements of Personality*—*Validity and Reliability of the GSOEP Big Five Inventory (BFI-S). J. Res. Personal..

[45] Heineck G. (2011). Does It Pay to Be Nice? Personality and Earnings in the United Kingdom. ILR Rev..

[46] Scott L.M., Janikas M.V., Fischer M.M., Getis A. (2010). Spatial Statistics in ArcGIS. Handbook of Applied Spatial Analysis.

[47] Johnston K., Ver Hoef J.M., Krivoruchko K., Lucas N., Johnston K., Ver Hoef J.M., Krivoruchko K., Lucas N. (2001). Using ArcGIS Geostatistical Analyst.

[48] Guth P.L. (2006). Geomorphometry from SRTM. Photogramm Eng Remote Sens..

[49] Talhelm T., Zhang X., Oishi S., Shimin C., Duan D., Lan X., Kitayama S. (2014). Large-Scale Psychological Differences Within China Explained by Rice Versus Wheat Agriculture. Science.

[50] Thompson B. (2002). What Future Quantitative Social Science Research Could Look Like: Confidence Intervals for Effect Sizes. Educ. Res..

[51] Vrieze S.I. (2012). Model Selection and Psychological Theory: A Discussion of the Differences between the Akaike Information Criterion (AIC) and the Bayesian Information Criterion (BIC). Psychol. Methods.

[52] Strobl C., Malley J., Tutz G. (2009). An Introduction to Recursive Partitioning: Rationale, Application, and Characteristics of Classification and Regression Trees, Bagging, and Random Forests. Psychol. Methods.

[53] Xu L., Wen X., Shi J., Li S., Xiao Y., Wan Q., Qian X. (2021). Effects of Individual Factors on Perceived Emotion and Felt Emotion of Music: Based on Machine Learning Methods. Psychol. Music.

[54] Kahng A.B., Mantik S., Markov I.L. (2002). Min-Max Placement for Large-Scale Timing Optimization. Proceedings of the 2002 International Symposium on Physical Design—ISPD ’02.

[55] Steiger J.H. (1980). Tests for Comparing Elements of a Correlation Matrix. Psychol. Bull..

[56] Fulmer C.A., Gelfand M.J., Kruglanski A.W., Kim-Prieto C., Diener E., Pierro A., Higgins E.T. (2010). On “Feeling Right” in Cultural Contexts: How Person-Culture Match Affects Self-Esteem and Subjective Well-Being. Psychol. Sci..

[57] Rentfrow P.J., Jokela M., Church T. (2017). Regional Differences in Personality: Causes and Consequences. The Praeger Handbook of Personality Across Cultures.

[58] Vuong Q.H., Napier N.K. (2015). Acculturation and Global Mindsponge: An Emerging Market Perspective. Int. J. Intercult. Relat..

[59] Liu C. (2018). Poverty Alleviation in Panhe. Times Rep. (Rush).

[60] Erma Y. Love in the Mountains (Shan Li Qing). https://y.qq.com/n/ryqq/songDetail/000WljIb2PxU7I.

[61] Nederhof A.J. (1985). Methods of Coping with Social Desirability Bias: A Review. Eur. J. Soc. Psychol..

[62] Stuetzer M., Audretsch D.B., Obschonka M., Gosling S.D., Rentfrow P.J., Potter J. (2018). Entrepreneurship Culture, Knowledge Spillovers and the Growth of Regions. Reg. Stud..

[63] Vuong Q.-H., Bui Q.-K., La V.-P., Vuong T.-T., Nguyen V.-H.T., Ho M.-T., Nguyen H.-K.T., Ho M.-T. (2018). Cultural Additivity: Behavioural Insights from the Interaction of Confucianism, Buddhism and Taoism in Folktales. Palgrave Commun..

[64] Rentfrow P.J., Gosling S.D., Potter J. (2008). A Theory of the Emergence, Persistence, and Expression of Geographic Variation in Psychological Characteristics. Perspect. Psychol. Sci..

[65] Carciofo R., Yang J., Song N., Du F., Zhang K. (2016). Psychometric Evaluation of Chinese-Language 44-Item and 10-Item Big Five Personality Inventories, Including Correlations with Chronotype, Mindfulness and Mind Wandering. PLoS ONE.

[66] John O.P., Naumann L.P., Soto C.J., John O.P., Robins R.W., Pervin L.A. (2008). Paradigm shift to the integrative Big Five trait taxonomy: History, measurement, and conceptual issues. Handbook of Personality: Theory and Research.

[67] Garretsen H., Stoker J.I., Soudis D., Martin R., Rentfrow J. (2019). The Relevance of Personality Traits for Urban Economic Growth: Making Space for Psychological Factors. J. Econ. Geogr..

[68] Vuong Q.-H., Ho M.-T., Nguyen H.-K.T., Vuong T.-T., Tran T., Hoang K.-L., Vu T.-H., Hoang P.-H., Nguyen M.-H., Ho M.-T. (2020). On How Religions Could Accidentally Incite Lies and Violence: Folktales as a Cultural Transmitter. Palgrave Commun..

