# Supplementary material for: Human Personality Is Associated with Geographical Environment in Mainland China
Source: Int J Environ Res Public Health. 2022 Aug 30;19(17):10819. doi: 10.3390/ijerph191710819 (PMC9517826; doi:10.3390/ijerph191710819)
Supplement: Supplementary file 1 [file ijerph-19-10819-s001.zip › ijerph-1867590-supplementary.pdf]

Contents

Table S1. Results from Multilevel Modelling for Agreeableness and the Elevation Coefficient of Variation. 2

Table S2. Results from Multilevel Modelling for Extraversion and the Elevation Coefficient of Variation. 3

Table S3. Results from Multilevel Modelling for Conscientiousness and the Elevation Coefficient of Variation. 4

Table S4. Results from Multilevel Modelling for Neuroticism and the Elevation Coefficient of Variation. 5

Table S5. Results from Multilevel Modelling for Openness and the Elevation Coefficient of Variation. 6

Table S6. Results of Parameter Search for the Prediction Models. 7

Table S7. Results from Multilevel Modelling for the Mean Elevation. 8

Table S8. Results from Multilevel Modelling for the Standard Deviation in Elevation. 9

Table S1. Results from Multilevel Modelling for Agreeableness and the Elevation Coefficient of Variation.

Predictor	Model 1	Model 2	Model 3	Model 4
	***β* (*p*) [95% CI]**	***β* (*p*) [95% CI]**	***β* (*p*) [95% CI]**	***β* (*p*) [95% CI]**
Sex	0.2092 (<0.001) [0.1456, 0.2728]	-0.1134 (<0.001) [-0.1573, -0.0695]	0.1901(<0.001) [0.1284, 0.2518]	-0.1215 (<0.001) [-0.1652, -0.0777]
Age	0.1420 (<0.001) [0.1410, 0.1430]	0.0231 (<0.001) [0.0217, 0.0246]	0.1271 (<0.001) [0.1259, 0.1283]	0.0232 (<0.001) [0.0217, 0.0247]
Education	1.4277 (<0.001) [1.4109, 1.4444]	0.1727 (<0.001) [0.1549, 0.1906]	1.2473 (<0.001) [1.2292, 1.2654]	0.1797 (<0.001) [0.1619, 0.1976]
Latitude		0.0633 (<0.001) [0.0622, 0.0644]		0.0638 (<0.001) [0.0627, 0.0649]
Longitude		0.0691 (<0.001) [0.0683, 0.0698]		0.0717 (<0.001) [0.0709, 0.0726]
Elevation CV			1.5359 (<0.001) [1.4671, 1.6047]	-0.3862 (<0.001) [-0.4414, -0.3309]
**Model fit statistics**				
AIC	146,037	123,649	144,183	123,465
BIC	146,078	123,708	144,232	123,531

Elevation CV: The elevation coefficient of variation; AIC: Akaike information criterion; BIC: Bayesian information criterion.

Table S2. Results from Multilevel Modelling for Extraversion and the Elevation Coefficient of Variation.

**Predictor**	**Model 1**	**Model 2**	**Model 3**	**Model 4**
	β **(*p*) [95% CI]**	***β*** **(*p*) [95% CI]**	***β*** **(*p*) [95% CI]**	***β*** **(*p*) [95% CI]**
Sex	0.5269 (<0.001) [0.4612, 0.5926]	0.2389 (<0.001) [0.1874, 0.2904]	0.5089 (<0.001) [0.4448, 0.5729]	0.2333 (<0.001) [0.1818, 0.2848]
Age	0.1242 (<0.001) [0.1231, 0.1253]	0.0193 (<0.001) [0.0176, 0.0210]	0.1101 (<0.001) [0.1089, 0.1114]	0.0193 (<0.001) [0.0176, 0.0211]
Education	1.1815 (<0.001) [1.1642, 1.1987]	0.0745 (<0.001) [0.0536, 0.0955]	1.0114 (<0.001) [0.9927, 1.0302]	0.0794 (<0.001) [0.0584, 0.1004]
Latitude		0.0542 (<0.001) [0.0529, 0.0555]		0.0545 (<0.001) [0.0532, 0.0558]
Longitude		0.0616 (<0.001) [0.0607, 0.0625]		0.0634 (<0.001) [0.0624, 0.0644]
Elevation CV			1.4479 (<0.001) [1.3765, 1.5193]	-0.2662 (<0.001) [-0.3313, -0.2012]
**Model fit statistics**				
AIC	147,949	133,244	146,412	133,181
BIC	147,991	133,302	146,462	133,248

Elevation CV: The elevation coefficient of variation; AIC: Akaike information criterion; BIC: Bayesian information criterion.

Table S3. Results from Multilevel Modelling for Conscientiousness and the Elevation Coefficient of Variation.

Predictor	Model 1	Model 2	Model 3	Model 4
	***β* (*p*) [95% CI]**	***β* (*p*) [95% CI]**	***β* (*p*) [95% CI]**	***β* (*p*) [95% CI]**
Sex	0.4998 (<0.001)	0.1908 (<0.001)	0.4823 (<0.001)	0.1805 (<0.001)
	[0.4352, 0.5645]	[0.1430, 0.2386]	[0.4192, 0.5454]	[0.1329, 0.2281]
Age	0.1471 (<0.001)	0.0357 (<0.001)	0.1334 (<0.001)	0.0358 (<0.001)
	[0.1460, 0.1481]	[0.0341, 0.0373]	[0.1322, 0.1346]	[0.0342, 0.0374]
Education	1.3100 (<0.001)	0.1352 (<0.001)	1.1447 (<0.001)	0.1442 (<0.001)
	[1.2931, 1.3270]	[0.1157, 0.1546]	[1.1262, 1.1632]	[0.1248, 0.1636]
Latitude		0.0559 (<0.001)		0.0565 (<0.001)
		[0.0547, 0.0570]		[0.0553, 0.0577]
Longitude		0.0660 (<0.001)		0.0695 (<0.001)
		[0.0652, 0.0668]		[0.0685, 0.0704]
Elevation CV			1.4082 (<0.001)	-0.4951 (<0.001)
			[1.3378, 1.4785]	[-0.5552, -0.4349]
**Model fit statistics**				
AIC	147,008	128,763	145,509	128,506
BIC	147,050	128,822	145,558	128,573

Elevation CV: The elevation coefficient of variation; AIC: Akaike information criterion; BIC: Bayesian information criterion.

Table S4. Results from Multilevel Modelling for Neuroticism and the Elevation Coefficient of Variation.

**Predictor**	**Model 1**	**Model 2**	**Model 3**	**Model 4**
	β **(*p*) [95% CI]**	***β*** **(*p*) [95% CI]**	***β*** **(*p*) [95% CI]**	***β*** **(*p*) [95% CI]**
Sex	-0.2775 (<0.001) [-0.3465, -0.2085]	-0.5942 (<0.001) [-0.6468, -0.5416]	-0.2943 (<0.001) [-0.3620, -0.2267]	-0.6071 (<0.001) [-0.6594, -0.5548]
Age	0.1129(<0.001) [0.1118, 0.1141]	-00.0015 (0.105) [-0.0032, 0.0003]	0.0998 (<0.001) [0.0985, 0.1011]	-0.0013 (=0.136) [-0.0031, 0.0004]
Education	1.1045 (<0.001) [1.0864, 1.1226]	-0.1023 (<0.001) [-0.1237, -0.0809]	0.9455 (<0.001) [0.9256, 0.9653]	-0.0911 (<0.001) [-0.1124, -0.0698]
Latitude		0.0577 (<0.001) [0.0564, 0.0590]		0.0585 (<0.001) [0.0572, 0.0598]
Longitude		0.0677 (<0.001) [0.0667, 0.0686]		0.0720 (<0.001) [0.0710, 0.0730]
Elevation CV			1.3541 (<0.001) [1.2787, 1.4295]	-0.6180(<0.001) [-0.6841, -0.5519]
**Model fit statistics**				
AIC	150,860	134,444	149,648	134,112
BIC	150,902	134,503	149,698	134,179

Elevation CV: The elevation coefficient of variation; AIC: Akaike information criterion; BIC: Bayesian information criterion.

Table S5. Results from Multilevel Modelling for Openness and the Elevation Coefficient of Variation.

Predictor	Model 1	Model 2	Model 3	Model 4
	***β* (*p*) [95% CI]**	***β* (*p*) [95% CI]**	***β* (*p*) [95% CI]**	***β* (*p*) [95% CI]**
Sex	0.7506 (<0.001) [0.6777, 0.8236]	0.4672 (<0.001) [0.4064, 0.5280]	0.7371 (<0.001) [0.6650, 0.8092]	0.4529 (<0.001) [0.3923, 0.5134]
Age	0.1019 (<0.001) [0.1007, 0.1031]	-0.0022 (<0.05) [-0.0043, -0.0002]	0.0914 (<0.001) [0.0899, 0.0928]	-0.0021 (=0.044) [-0.0041, -0.0001]
Education	1.3317 (<0.001) [1.3125, 1.3508]	0.2323 (<0.001) [0.2076, 0.2571]	1.2041 (<0.001) [1.1830, 1.2253]	0.2448 (<0.001) [0.2201, 0.2695]
Latitude		0.0550 (<0.001) [0.0535, 0.0565]		0.0559 (<0.001) [0.0544, 0.0574]
Longitude		0.0607 (<0.001) [0.0596, 0.0617]		0.0654 (<0.001) [0.0643, 0.0666]
Elevation CV			1.0860 (<0.001) [1.0056, 1.1663]	-0.6854 (<0.001) [-0.7619, -0.6089]
**Model fit statistics**				
AIC	154.164	143,130	153,473	142,825
BIC	154,206	143,188	153,523	142,892

Elevation CV: The elevation coefficient of variation; AIC: Akaike information criterion; BIC: Bayesian information criterion.

Table S6. Results of Parameter Search for the Prediction Models.

**Parameters**	**Agreeableness**	**Extraversion**	**Conscientiousness**	**Neuroticism**	**Openness**
n_estimators	125	163	151	155	111
max_depth	5	7	5	5	5
min_samples_leaf	37	52	25	33	3
min_samples_split	10	37	22	5	8
max_features	1	0.5	0.8	0.7	0.5

Table S7. Results from Multilevel Modelling for the Mean Elevation.

**Predictor**	**Agreeableness**	**Extraversion**	**Conscientiousness**	**Neuroticism**	**Openness**
	**β (p) [95% CI]**	**β (p) [95% CI]**	**β (p) [95% CI]**	**β (p) [95% CI]**	**β (p) [95% CI]**
Sex	-0.1631(<0.001)	0.1865 (<0.001)	0.1307 (<0.001)	-0.6577 (<0.001)	0.3974 (<0.001)
	[-0.2059, -0.1203]	[0.1359, 0.2370]	[0.0844, 0.1770]	[-0.7087, -0.6066]	[0.3382, 0.4567]
Age	0.0215 (<0.001)	0.0175 (<0.001)	0.0337 (<0.001)	-0.0036 (<0.001)	-0.0046 (<0.001)
	[0.0200, 0.0229]	[0.0158, 0.0192]	[0.0321, 0.0352]	[-0.0053, -0.0019]	[-0.0066, -0.0026]
Education	0.1817 (<0.001)	0.0840 (<00.001)	0.1460 (<0.001)	-0.0909 (<0.001)	0.2449 (<0.001)
	[0.1643, 0.1991]	[0.0635, 0.1045]	[0.1272, 0.1649]	[-0.1116, -0.0701]	[0.2208, 0.2690]
Latitude	0.0634 (<0.001)	0.0543 (<00.001)	0.0560 (<0.001)	0.0578 (<0.001)	0.0551 (<0.001)
	[0.0623, 0.0644]	[0.0530, 0.0555]	[0.0548, 0.0571]	[0.0566, 0.0591]	[0.0537, 0.0566]
Longitude	0.0662 (<0.001)	0.0586 (<0.001)	0.0625 (<0.001)	0.0640 (<0.001)	0.0566 (<0.001)
	[0.0654, 0.0669]	[0.0577, 0.0594]	[0.0617, 0.0634]	[0.0631, 0.0649]	[0.0556, 0.0577]
Elevation	0.0005 (<0.001)	0.0006 (<0.001)	0.0006 (<0.001)	0.0007 (<0.001)	0.0007 (<0.001)
Mean	[0.0005, 0.0005]	[0.0005, 0.0006]	[0.0006, 0.0007]	[0.0006, 0.0007]	[0.0007, 0.0008]
**Model fit statistics**					
AIC	122,051	131,960	126,776	132,622	141,490
BIC	122,118	132,026	126,843	132,689	141,556

Elevation Mean: The mean value of elevation; AIC: Akaike information criterion; BIC: Bayesian information criterion;

0^b: This parameter is redundant, as a result, it was set as zero.

Table S8. Results from Multilevel Modelling for the Standard Deviation in Elevation.

**Predictor**	**Agreeableness**	**Extraversion**	**Conscientiousness**	**Neuroticism**	**Openness**
	β **(*p*) [95% CI]**	***β*** **(*p*) [95% CI]**	***β*** **(*p*) [95% CI]**	***β*** **(*p*) [95% CI]**	***β*** **(*p*) [95% CI]**
Sex	-0.1521 (<0.001)	0.1953 (<0.001)	0.1439 (<0.001)	-0.6422 (<0.001)	0.4163 (<0.001)
	[-0.1950, -0.1092]	[0.1448, 0.2458]	[0.0974, 0.1904]	[-0.6936, -0.5908]	[0.3567, 0.4760]
Age	0.0214 (<0.001)	0.0173 (<0.001)	0.0336 (<0.001)	-0.0036 (<0.001)	-0.0045 (<0.001)
	[0.0199, 0.0228]	[0.0156, 0.0190]	[0.0320, 0.0351]	[-0.0054, -0.0019]	[-0.0065, -0.0025]
Education	0.1790 (<0.001)	0.0816 (<0.001)	0.1428 (<0.001)	-0.0945 (<0.001)	0.2406 (<0.001)
	[0.1615, 0.1964]	[0.0611, 0.1021]	[0.1239, 0.1617]	[-0.1154, -0.0736]	[0.2163, 0.2648]
Latitude	0.0638 (<0.001)	0.0548 (<0.001)	0.0565 (<0.001)	0.0584 (<0.001)	0.0557 (<0.001)
	[0.0627, 0.0648]	[0.0535, 0.0560]	[0.0553, 0.0576]	[0.0571, 0.0596]	[0.0542, 0.0572]
Longitude	0.0655 (<0.001)	0.0575 (<0.001)	0.0617 (<0.001)	0.0632 (<0.001)	0.0560 (<0.001)
	[0.0647, 0.0662]	[0.0566, 0.0584]	[0.0608, 0.0625]	[0.0623, 0.0641]	[0.0549, 0.0570]
Elevation	0.0011 (<0.001)	0.0012 (<0.001)	0.0013 (<0.001)	0.0013 (<0.001)	0.0014 (<0.001)
STD	[0.0010, 0.0011]	[0.0011, 0.0013]	[0.0012, 0.0013]	[0.0012, 0.0014]	[0.0013, 0.0015]
**Model fit statistics**					
AIC	122,285	131,989	127,063	132,978	141,906
BIC	122,351	132,055	127,129	133,044	141,972

Elevation STD: The standard deviation in elevation; AIC: Akaike information criterion; BIC: Bayesian information criterion.
